# Achievements and gaps in projection studies on the temperature-attributable health burden: Where should we be headed?

**DOI:** 10.3389/fepid.2022.1063871

**Published:** 2022-12-16

**Authors:** Masna Rai, Susanne Breitner, Siqi Zhang, Ana G. Rappold, Alexandra Schneider

**Affiliations:** ^1^ Institute of Epidemiology, Helmholtz Center Munich, Neuherberg, Germany; ^2^ Institute for Medical Information Processing, Biometry, and Epidemiology, LMU Munich, Munich, Germany; ^3^ Center for Public Health and Environmental Assessment, Office of Research and Development, United States Environmental Protection Agency, Research Triangle Park, Durham, NC, United States

**Keywords:** climate change, projection studies, health burden, review, gaps

## Abstract

Future projection of the temperature-related health burden, including mortality and hospital admissions, is a growing field of research. These studies aim to provide crucial information for decision-makers considering existing health policies as well as integrating targeted adaptation strategies to evade the health burden. However, this field of research is still overshadowed by large uncertainties. These uncertainties exist to an extent in the future climate and population models used by such studies but largely in the disparities in underlying assumptions. Existing studies differ in the factors incorporated for projection and strategies for considering the future adaptation of the population to temperature. These differences exist to a great degree because of a lack of robust evidence as well as gaps in the field of climate epidemiology that still require extensive input from the research community. This narrative review summarizes the current status of projection studies of temperature-attributable health burden, the guiding assumptions behind them, the common grounds, as well as the differences. Overall, the review aims to highlight existing evidence and knowledge gaps as a basis for designing future studies on temperature-attributable health burden estimation. Finding a robust methodology for projecting the future health burden could be a milestone for climate epidemiologists as this would largely benefit the world when applying this technique to project the climate-attributable cause-specific health burden and adapt our existing health policies accordingly.

## Introduction

Projection studies estimate the future health burden directly or indirectly caused by the changing climate. These studies, giving us a future picture of the climate-attributable health burden, are crucial in that they urge stakeholders, policymakers, civil society, scientists, and the public to practice and enforce mitigation measures for climate protection. *Mitigation*, as defined by the fourth assessment report of the Intergovernmental Panel on Climate Change (IPCC), is an “anthropogenic intervention to reduce the sources or enhance the sinks of greenhouse gases” (1). Management of the climate crisis through climate change mitigation seemed hopeful until recently, when the recent IPCC report was released. The report states that unless there are immediate large-scale mitigation measures to reduce greenhouse gas emissions, it is beyond reach to limit global warming to 1.5°C or even 2°C (2). Therefore, mitigation is not enough to combat the harms of the rapidly changing climate. Amidst this crisis, adaptation strategies can help to build climate resilience. The IPCC report defines *adaptation* as “adjustment in natural or human systems in response to actual or expected climatic stimuli or their effects, which moderates harm or exploits beneficial opportunities” (1).

The goal of today is to build a climate-resilient society that can be possible only in the presence of efficient adaptation strategies in addition to mitigation measures. For this, evidence-based planning of health policies and adaptation measures need to be designed by public health professionals, implemented by health authorities, and incorporated by society. In this regard, studies projecting the climate-attributable future health burden can support laying a foundation of evidence and aid in planning effective adaptation strategies. Projection studies help in the planning of adaptation strategies in the following ways:

i. They estimate the future climate-related health burden, allowing for the planning of healthcare resources.
ii. They estimate the climate-related health burden for different causes, which enables us to focus on adaptation plans for specific diseases or health outcomes.
iii. They estimate the climate-related health burden for different population subgroups, which enables us to identify those who are especially at risk of climate change, allowing us to target and adapt our adaptation policies for the vulnerable and susceptible population groups.

Projection studies emerged during the late 1900s (3, 4). Early studies projected temperature-related deaths for 2020 and 2050 for selected cities. During the 2000s, research in the field started growing (5–10); however, studies focused on North America (5–8) and Europe (8–10). After 2010, climate epidemiology started being prioritized, and projection studies were expanding (11–21), with research still focused on the US and Europe. In the mid-2010s, projection studies started in China (22–29) and Latin America (30), while projection studies in the US, Europe, and Australia continued to expand (31–44). The era of 2010 was a remarkable period for climate epidemiology, not only because of the expanding field with large epidemiological studies incorporating methodological advancements but also because projection on other critical aspects related to climate change, apart from the previously explored temperature-related total mortality, was initiated. Researchers started exploring the burden of cardiovascular and respiratory diseases (27), vector-borne diseases like Malaria (45), and sensitive issues like children's health were highlighted (46).

The field of climate-attributable health impact research is expanding rapidly, however, still overshadowed by large uncertainties and differing largely in their guiding principles. These differences exist to a great degree because of a lack of robust evidence as well as gaps in the field of climate epidemiology that still require extensive input from the research community. In this context, the research community would largely benefit from a review that summarizes current status, assumptions, and evidences, which would facilitate the planning of future studies. Existing reviews of the field either focused only on heat-related mortality (47) or climate change mitigation outcomes (48). This narrative review, therefore, aims to summarize the current status of projection studies of temperature-attributable health burden, the guiding assumptions behind them, the common grounds, as well as the differences. Overall, the review aims to highlight existing evidence and knowledge gaps as a basis for designing future studies on temperature-attributable health burden estimation.

## Temperature attributable health burden and earlier misconceptions

The results from early projection studies partly raised misconceptions in that climate change looked beneficial. For example, the study by Martens et al., which included various cities from around the world, found for most cities that climate change is likely to cause a reduction in mortality rates due to decreasing winter mortality. The study claimed this effect was more pronounced for cardiovascular mortality in older people in cities with temperate or cold climates at present (4). In addition, the result of the study was not generalizable to other regions of the world with different climatic conditions. Conversely, another study by Kalkstein et al., projecting mortality in US cities for 2020 and 2050, found summer mortality to increase dramatically while winter mortality to decrease slightly, as a result of climate change (3), illustrating that the net impact of climate change would be more harmful than beneficial. Nevertheless, this study was done in a single country and needed validation by a larger study across regions of varying climatic and socio-economic conditions.

From these studies, it was evident that there exists a temperature-related health burden in association with future temperatures, with losses from heat-related deaths on the one hand and benefits from cold-related deaths on the other. Therefore, for valid future projections, studies were needed that estimated the net future temperature-related burden incorporating both heat and cold impacts. A 2011 study by Ballester et al. (14) systematically estimated the heat- and cold-related deaths in 200 European regions. The results showed that the rise in deaths from heat would start to compensate completely the reduction of cold-related mortality during the second half of the 21st century. This study provided evidence that climate change would not be beneficial in the long run, at least for the European regions included in the study. To validate the results, the climate epidemiology community needed a large study investigating such associations across regions with varying climatic and socio-economic conditions. In 2017, a multi-country and multi-city study by Gasparrini et al. (49) projected the net temperature-related health burden in 451 locations from 23 countries. This study is one of the most comprehensive studies in terms of including cities from around the world in a single study. The results were seen to vary across regions. In temperate areas such as northern Europe, East Asia, and Australia, the less intense warming and the large decrease in cold-related deaths would induce a null or marginally negative net effect. In contrast, warmer regions, including central and southern America, Europe, and Southeast Asia, would expect steep increases in heat-related mortality resulting in a large net burden. The study concluded that the negative health impacts of climate change would disproportionately affect warmer regions of the world, and regions lagging in infrastructures and technology. From this observation, it is clear that people worldwide are vulnerable to climate change—but not equally. Nevertheless, it is essential to note that this study did not account for influential factors like differing health effects of heat or cold across different population age groups and changing demographic structures over time (i.e., population aging). Therefore, the observed decrease in the net burden in temperate first-world nations might have been rather biased.

## Fundamental concepts: Exposure response functions

**Figure 1:**
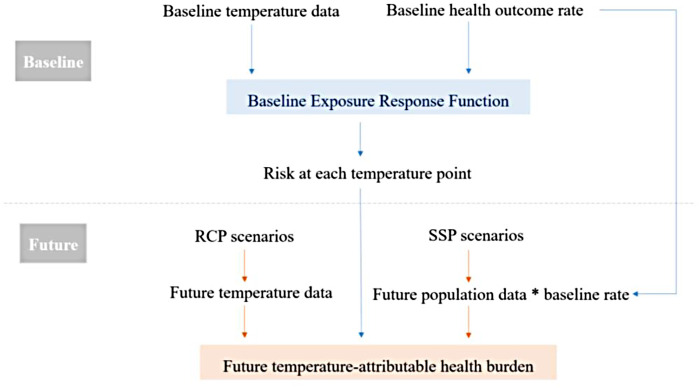
A flowchart of the standard methodology of temperature-attributable health outcome projections.

Future projections of the health burden are primarily based on present-day observations. Studies usually start with time-series data of health outcome during a reference baseline period to explore the association between temperature and the health outcome of interest (49, 50). This association is often termed *Exposure Response Function* (ERF). From the ERFs, the risk of the health outcome at each temperature point is obtained, which is then extrapolated to the future temperature observations (50). Researchers obtain future temperature data from climate modelers, estimate the future risk under these temperature projections and quantify the differences in the health burden in the future compared to the baseline. Figure 1 summarizes the standard practiced methodology of temperature-attributable health outcome projection.

Until recent years, most studies have applied an overall baseline ERF for future projections, assuming all population subgroups to act similarly to a given temperature (49). However, this approach underestimates the future health burden as the most vulnerable and susceptible population subgroups, like the elderly, are assumed to have the same baseline rate of risk as the younger population. A study by Rai et al. elaborates on this drawback of using an overall ERF by projecting future temperature-related total mortality burden by applying two frameworks; an overall ERF and age-specific ERFs (42). The results show a considerable underestimation of the health burden when not considering the age-specific ERFs. Therefore, projection studies incorporating age-specific ERFs might provide a more valid estimation of the future health burden (19, 51–53).

Nonetheless, all the above principles of projection studies assume that the ERFs of the future population remain constant as the present-day ERF, i.e., no adaptation of the human body to the changing climate occurs. This might introduce large biases. So far, few studies have considered population adaptation when estimating the future temperature-related health burden (5, 6, 8, 9, 54–57). These studies differ in their approaches. Some of the earlier approaches used ERFs of analogous summers or cities for future projections (5, 6); for example, a test city was assumed to be similar to a larger reference city in the future. For present-day large cities, some harsh summers with a temperature distribution similar to the modelled future temperature were selected as the reference summer. The population of the test city was then assumed to react to temperature increases in the same way as the population from the reference city or the reference summer in the future. However, these approaches were largely based on untestable assumptions, resulting in large uncertainties. More recent studies assume population acclimatization over a few degrees (8, 9) or a shift in the ERF between temperature and health outcomes (50). However, there is no established general methodological procedure to account for physiological adaptation to changing climate.

## Scenarios

Projecting health burden is estimating health outcomes under uncertainty in a number of systems including the environmental, human, and socio-economic systems, and the complex interaction between them. For this reason, climate change research has been working with future scenarios, which include a set of climatic and socio-economic assumed conditions that we might experience in the future.

Earlier projection studies estimated the health burden under different climate scenarios, i.e., the Representative Concentration Pathways (RCPs) (19, 38, 49, 51, 52, 58). While these studies addressed climate uncertainty and some aspects of population and economic changes incorporated within the RCPs, they did not account for other possible changes in societal factors such as demographics, human development (for example, health and education), economic growth, inequality, governance, technological change, and policy orientations. All these factors are considered by the different scenarios under the Shared Socio-Economic Pathways (SSPs) (59). Comparatively few studies have considered the SSP scenarios when estimating the future temperature-related health burden (42, 53). Although practiced, using a combination of all four RCPs (60) and five SSPs (61) was not the most efficient and convincing methodology because many RCP-SSP combinations seem implausible. A publication by O'Neill et al., explains the plausibility of various RCP-SSP scenario combinations (62). For example, the combination of RCP 8.5 (the worst climate change scenario) and SSP1 (the scenario with lowest challenge to adaptation and mitigation) seems implausible. In 2021, IPCC revised the RCPs and released an update of the climate scenarios integrating the plausible SSP scenarios into the RCPs, termed SSP- RCP scenarios (2).

Apart from the RCP-SSP scenarios, a crucial aspect to be considered for a justifiable future projection is population aging. The SSP scenarios consider the change in population; however, like with the age-specific ERFs, a key aspect to be integrated is the age-specific population growth (demographic change), which had been ignored until recently (28, 29, 42). Not considering the increasing proportion of older people, especially in first-world nations, would lead to underestimating the climate-related health burden as this population subgroup is one of the most susceptible.

## Uncertainties and assumptions

The field of climate epidemiology dominated largely by uncertainties and assumptions. Some of the determining sources of uncertainties are the modelled future climate and societal scenarios, i.e., the RCPs and the SSPs. These scenarios, providing us with a range of plausible future scenarios, are largely based on assumptions. However, the efforts to continuously reevaluate and update these scenarios have helped in overcoming uncertainties (59). Table 1 lists the major sources of uncertainties and underlying assumptions.

**Table 1:** Summary of uncertainties and assumptions in climate-attributable health burden projection.

Sources of uncertainties	Underlying assumptions
Climate models	Assumptions made while defining various atmospheric parameters under future climate scenarios to obtain the future temperature data.
Population projections	Assumptions made while defining future demographics, human development (for example, health and education), economic growth, inequality, governance, technological change, and policy orientations under future socioeconomic scenarios to obtain the future population data.
Physiological adaptation of human body to the changing climate	Use of analogous cities or summers or changing the slope of the baseline ERFs.
Socioeconomic changes, technological advancements and changes in healthcare system and settings	Using GDP as a factor determining the adaptive capacity and using it as a factor potentially changing the temperature-related health risk.

## Gaps

Although the field of climate epidemiology is progressing rapidly, there still exist significant research gaps. The gaps have been listed and elaborated in the following sections.

### Focus on heat and not the entire temperature range

One of the largest needs for climate epidemiology research is to shift the focus from heat-related mortality projections to total temperature-related mortality projections, which include both heat- and cold-related mortality. Most earlier projection studies focused on heat-related mortality (5, 8, 16, 18, 22, 27, 40, 63), leaving behind the cold-related future attributable burden. However, it is important to consider that cold-related mortality is only minimally attributable to extreme cold but mostly to moderate cold or air temperature changes (temperature variability) that would persist in the future, even with a warming climate. Therefore, projection studies would not be complete without considering the cold-related mortality and estimating the net temperature-related mortality burden (64).

### Use of overall ERF rather than sub-group specific ERF

Another overshadowed aspect is the failure to incorporate the age-specific ERFs and the age-specific population growth rates when estimating the future temperature-related health burden. Although some recent studies considered this aspect (28, 29), these studies focused only on heat-related impacts and ignored the cold-related impacts. Only a handful of studies have considered age-specific ERFs and population growth rates to estimate the future net temperature-related health burden (42). Moreover, other climate vulnerability and susceptibility factors apart from age have been left entirely unaddressed.

### Considering a constant ERF

Majority of the projection studies consider constant response of the population to a given temperature. However, response of the human body to a given temperature might change in the future, leading to either adaptation or sensitivity. Only a small number of studies have considered physiological adaptation of the human body (9, 55, 57) to heat and no studies have considered the physiological changes in response to cold. As discussed above, cold-related mortality would continue to dominate a large fraction of temperature-related mortality. Therefore, projection studies would not be complete without considering the future changes in the cold-mortality relationships, i.e., taking into account also the adaptation or increasing sensitivity to cold in the future (64). Existing evidence on non-decreasing (65, 66) or even increasing cold effects (67) over time suggests that together with adaptation to heat, on the one hand, the future population might be increasingly susceptible to cold on the other (64). Furthermore, it is essential to note that physiological adaptation pathways cannot be generalized but need to be considered specifically for a population of interest. A large multi-country study investigating the temporal variation in the heat-mortality association has demonstrated that the adaptation pattern or heat sensitivity varies across locations (68).

### Lack of simultaneous consideration of socioeconomic adaptation

A crucial aspect not yet fully accounted for is the future population adaptation particularly in the context of social and economic inequalities. The future population is foreseen to undergo not just single but multiple simultaneous adaptation pathways (69). In addition to physiological adaptation, future infrastructure changes, technological advancements, and socio-economic challenges might play an important role in influencing how the human body reacts to temperature. Some recent studies (29) have explored this aspect of adaptation by defining future adaptive capacity as a factor of the future Gross Domestic Product (GDP). However, physiological and socioeconomic adaptation have not been yet considered simultaneously but rather independently. A recently proposed methodological framework for health burden projections aims to overcome this gap by systematically incorporating future physiological adaptation-sensitivity and socio-economic adaptive capacities as factors potentially changing the ERF in the future (69).

Future shifts in infrastructure, healthcare, as well as technological advancements might change the mortality rate. These might be changes in the overall mortality rate (70) or cause-specific mortality rates (71). Failure to incorporate these changes in studies estimating the future overall or cause-specific mortality might lead to overestimating the future temperature-related burden. Only a limited number of projection studies have so far incorporated expected changes in mortality rates while estimating the future temperature-related mortality burden (69).

### Focus only on specific health outcomes

Another major gap is the focus of projection studies on total mortality. Although recent studies also project cause-specific mortality under different ranges of future climate and population change scenarios (42, 44, 49, 53, 43), these studies are limited to specific regions. Furthermore, no studies have looked into other critical aspects like cause-specific hospitalizations.

### No studies in rural areas

In addition, all projection studies, including the largest multi-country study, have focused on cities (49) leaving behind the rural areas. It is yet unknown if rural areas might show different temperature effects in the future compared to cities or rather similar effects as, depending on location, exposure intensity, population structure, and susceptibility might be quite different. The results from the EU HORIZON2020 project EXHAUSTION show that temperature effects vary among European regions. Within this project, it was observed that the heat effects on mortality in Northern Europe were stronger in urban areas than that in rural areas, whereas, both heat and cold effects in the rural areas were found to be similar to that observed in cities in other parts of Europe (72). Extensive studies in other regions with different climatic and socio-economic conditions are required to verify this finding.

### Lack of representation

One of the largest gaps of projection studies is that they are limited to certain regions of the world, mostly North America, Europe, and East Asia. Other regions of the world, which might be facing the largest consequences of climate change (73), like Africa, South Asia, and the Middle East have been largely underrepresented. This issue also arises due to lack of data availability from those regions.

## Needs and recommendations

The following section lists and describes the needs and recommendations:

a) Inclusion and representativeness
  i. The climate epidemiology community needs more inclusive projection studies from across regions of diverse geographic, climatic, and socio-economic conditions.
  ii. Studies from rural areas and less urbanized areas are needed for a comprehensive understanding of climate-health association. In addition, studies projecting health burden at a finer geographical resolution with calibrated temperature models would be helpful for stakeholders in understanding and addressing the future risks at a community level.
b) Methodology
  i. Projection studies need to be designed to look at not just the heat- or cold-related burden separately, but a combined net temperature-related burden.
  ii. Future studies should incorporate sub-group specific ERFs (e.g., age-specific ERFs), rather than the overall ERFs, as they provide a reasonable and less biased estimation of the future health burden (19, 51–53).
  iii. Extensive baseline studies to explore susceptible and vulnerable population subgroups other than the elderly are recommended for a substantially valid projection of the future health burden. To achieve this, establishment of cohorts with all-encompassing individual characteristics and large enough to cover regions of varying climatic and socio-economic conditions is recommended.
  iv. The establishment of a standard procedure for accounting for future population adaptation is recommended.
c) For driving policy
  i. Extensive investigation on potential further adaptation factors which could be influenced by policy makers and stakeholders and health care provides or public health institutions is needed.

## Summary

Projection studies estimating the future climate-attributable health burden are crucial as they would aid in designing, adapting, and implementing targeted adaptation measures, as well as stressing the urgency of mitigation actions. This would help public health professionals in building a climate change resilient community.

Climate epidemiologists should focus on advancing projection studies but also on gathering extensive and unbiased baseline associations between temperature and cause-specific health outcomes, identifying the most vulnerable and susceptible population subgroups in regions with varying climatic and socio-economic conditions. As these baseline associations are the backbone of projection studies, researchers should focus on gathering valid and extensive baseline evidence.

**Figure 2:**
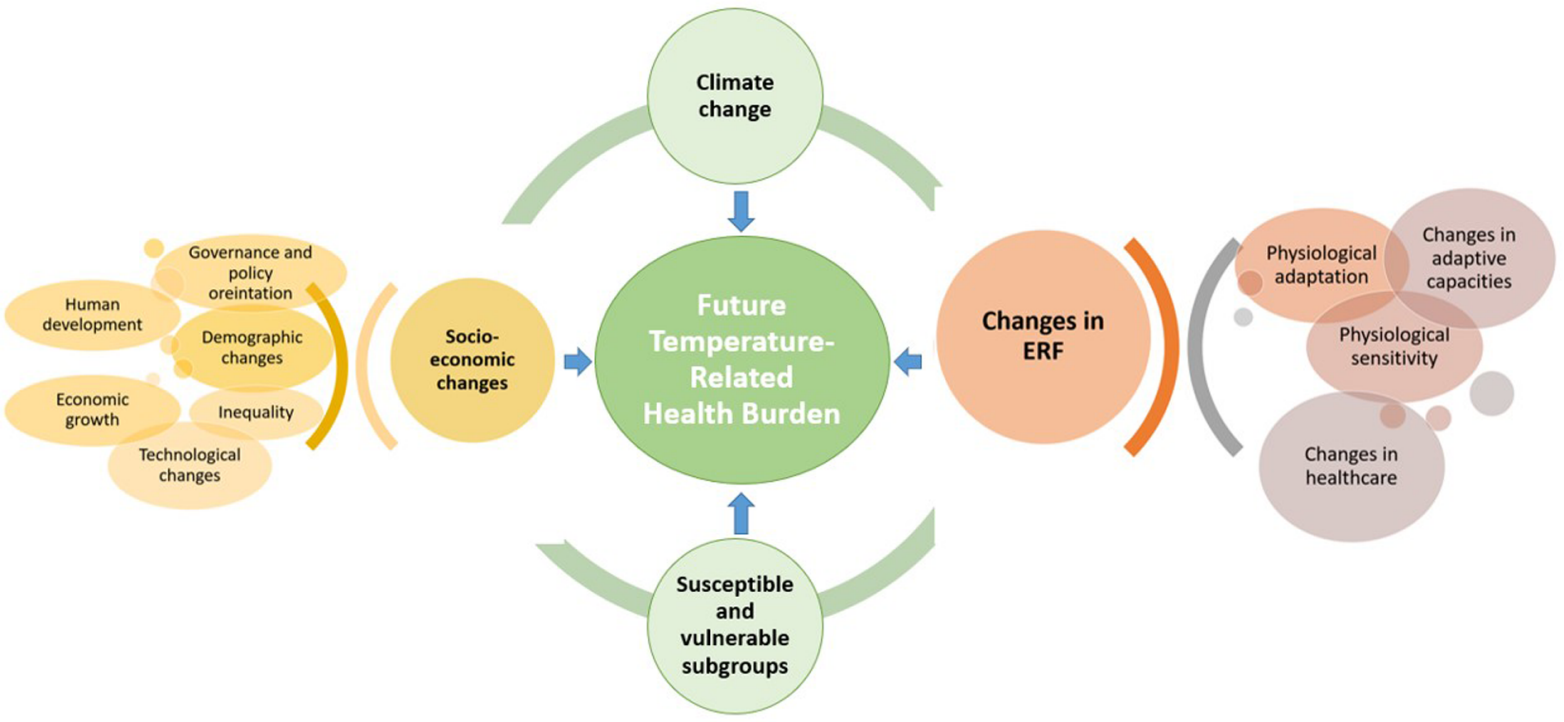
Dynamics influencing the future temperature-related health burden.

Further validation studies are required to establish a common framework and guidelines for future projection. Future studies in the field should focus on other health outcomes in addition to total mortality. The studies should attempt to estimate the net temperature-related health burden considering the subgroup-specific ERFs, future subgroup-specific population change, future mortality or hospital admission rates, and above all, the possible physiological and socio-economic adaptation. To summarize, future studies should account for all the complex dynamics, which play a role in determining temperature-related mortality (Figure 2).

## Author disclaimer

The research described in this article has been reviewed by the Center for Public Health and Environmental Assessment, EPA, and approved for publication. The contents of this article should not be construed to represent agency policy. The views expressed in this manuscript are those of the individual authors and do not necessarily reflect the views and policies of the U.S. Environmental Protection Agency. Mention of trade names or commercial products does not constitute endorsement or recommendation for use.
